# Primary neuroendocrine carcinoma of the fallopian tube with exceeding 10 years follow-up: Case report and review of the literature

**DOI:** 10.1016/j.gore.2025.101692

**Published:** 2025-02-11

**Authors:** Jun Ma, Yujing Tang, Wei He, Jun Shi

**Affiliations:** a Department of Obstetrics and Gynecology, Renji Hospital, Shanghai Jiao Tong University School of Medicine, Shanghai 200127 China; b Department of Pathology, Renji Hospital, Shanghai Jiao Tong University School of Medicine, Shanghai 200127 China

**Keywords:** Neuroendocrine carcinoma, Fallopian tube, Epithelial tumor

## Abstract

Neuroendocrine carcinomas typically originate from the digestive system and lungs; however, their occurrence in the female genital tract, particularly in the fallopian tube, is exceedingly rare. Currently, there is no established treatment guideline for neuroendocrine neoplasms of the fallopian tube. In this report, we presented a case of small cell neuroendocrine carcinoma of the fallopian tube, with a survival period exceeding 10 years—which is the longest recorded survival among reported cases of fallopian tube neuroendocrine tumors. The patient underwent a standard open radical tumor resection and lymphadenectomy, followed by adjuvant chemotherapy with cisplatin and etoposide, offering valuable evidence for potential standardized treatment approaches in such rare case.

## Highlights

• Neuroendocrine carcinoma of the fallopian tube is very rare.
• We present a neuroendocrine carcinoma of the fallopian tubes that have survived for over 10 years.
• We emphasized the effectiveness of surgery and EP chemotherapy for neuroendocrine carcinoma of the fallopian tubes.

## 1 Introduction

Neuroendocrine neoplasms (NEN) can occur in various parts of the body, most frequently in the lungs and gastrointestinal tract, but are relatively rare in the female reproductive tract, accounting for only 2 % of all gynecological malignancies. They are most commonly found in the ovary and cervix, with primary neuroendocrine tumors of the fallopian tube being extremely rare. According to the fifth edition of the World Health Organization (WHO) classification of female genital tumors, NENs are now divided into neuroendocrine tumors (NETs) and neuroendocrine carcinomas (NECs). NETs are further graded into grade 1 and grade 2 tumors, while NECs of the female genital tract are classified into small cell and large cell variants. To the best of our knowledge, only 7 prior cases of primary NEC and 5 cases of NET in the fallopian tube have been reported. Although the broad classification and histopathological features of neuroendocrine carcinomas (NECs) can appear similar across various sites in the female genital tract, there may be certain distinctions between fallopian tube NEC and ovarian NEC. Given that primary fallopian tube NEC is exceedingly rare, definitive diagnosis often hinges on identifying a distinct tumor mass arising from the fallopian tube itself, with secondary involvement of the ovary. Differentiating the primary site is crucial not only for accurate staging but also for understanding the disease’s clinical behavior, although surgical management and adjuvant chemotherapy typically remain similar to those used for ovarian NEC. Some neuroendocrine neoplasms can secrete hormones and produce hormone related symptoms. Neuroendocrine neoplasms mainly diagnosed through pathology and immunohistochemistry. Here, we present a new case, fully demonstrated its clinical manifestations, pathological features, and the longer clinical tumor survival after a comprehensive treatment of the tumor staging surgery and standardized postoperative chemotherapy.

## 2 Case history

**Fig. 1:**
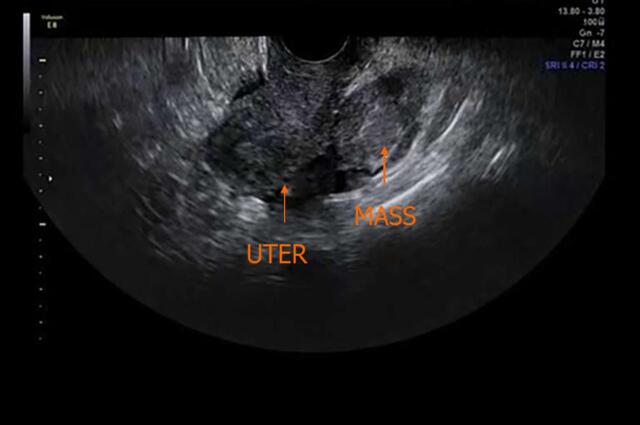
Image of ultrasound examinations.

**Fig. 2:**
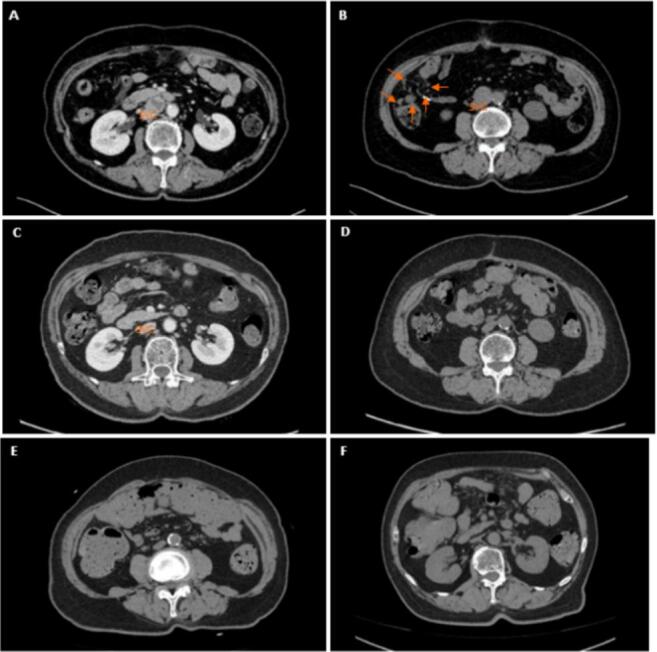
**Time axis of the patient’s detection, treatment, and post-operative follow-up of NEC.** A and B: Abdominal CT shows focus in the right abdominal cavity, posterior peritoneum, and pelvic cavity before surgery. C and D: Abdominal CT shows scattered lymph nodes in the retroperitoneum and iliac vessels are similar have significantly reduced after chemotherapy. E and F: Abdominal CT shows lymph nodes in retroperitoneal and bilateral iliac vascular is similar as before after 10 years. (SVC: superior vena cava).

A 70-year-old postmenopausal woman was referred to Renji Hospital, Shanghai Jiao Tong University School of Medicine due to irregular vaginal bleeding that had persisted for three months. She had a history of four pregnancies, resulting in two live births. Her family history was unremarkable. She had been diagnosed with hypertension three years prior and was taking Valsartan Capsules 80 mg daily. Pelvic examination revealed a mobile mass in the right adnexal region. Ultrasound examination showed a highly dense right adnexal mass measuring 41x62 x46 mm (Fig. 1). A computed tomography (CT) scan of the chest was normal. Abdominal CT showed a 6 cm lesion arising from the right adnexa with no obvious enlarged lymph nodes（Fig. 2 A-B）. Routine laboratory tests indicated that the serum level of CA-125 was 97.4 U/ml (normal range: 0–35 U/ml), CEA was 5.55 ng/ml (normal range: 0–5 ng/ml), CA-199 was 34.5 U/ml (normal range: 0–35 U/ml), and AFP was 2.1 ng/ml (normal range: 0–9 ng/ml). Both CA-199, Alpha-Fetoprotein (AFP) and neuron-specific enolase (NSE) levels were within normal limits. The patient exhibited no hormone secretion-related clinical symptoms, such as carcinoid syndrome or ACTH overproduction.

**Fig. 3:**
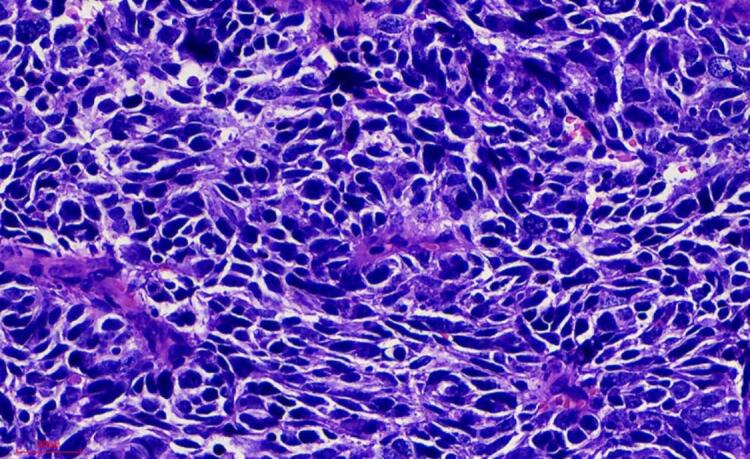
Dense monomorphic population of undifferentiated cells. (H&Ex400).

A radical surgery via laparotomy was performed. On laparotomy, 150 ml ascitic fluid was found in the pelvic cavity. The right fallopian tube was thickened, measuring approximately 61x50 x43 mm. A cauliflower-like tumor, approximately 3 cm in diameter, was present at the right fimbriated extremity of the fallopian tube. There was dissemination of tumor on the abdominal and retroperitoneal bilateral sacral ligament, as well as the splenic flexure, hepatic flexure side of the peritoneum, and bowel surfaces. A simple total hysterectomy (TH), bilateral salpingo-oophorectomy (BSO), and omentectomy were performed. Histopathologic examination revealed poorly differentiated small cell neuroendocrine carcinoma of the right fallopian tube (Fig. 3), with cancerous tissue also involving the right ovary. Metastases of poorly differentiated adenocarcinoma were observed in the greater omentum, while the peritoneal washing was negative. There were no metastases to the uterus or opposite ovary and fallopian tube The overall pathologic stage was T3NxM0, FIGO stage IIIC.

Importantly, intraoperative findings demonstrated that the tumor mass centered around the fimbrial end of the right fallopian tube and extended superficially onto the right ovary, rather than arising de novo from ovarian tissue. Histopathologic sections further supported the fallopian tube as the primary site, with secondary ovarian involvement. This distinction was critical to our final diagnosis of primary fallopian tube NEC.

**Fig. 4:**
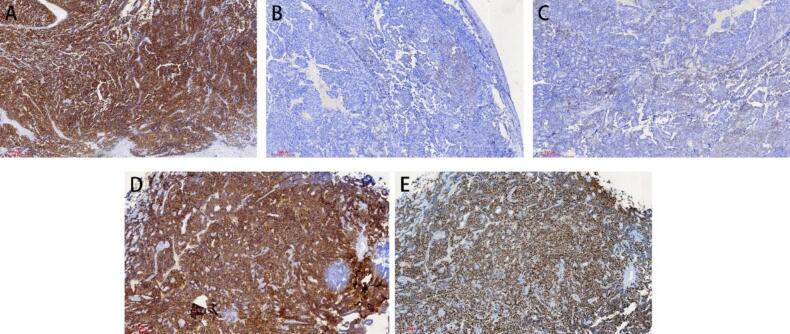
Homogeneous positive immunostaining A. SYN expression (IHC) B. CgA expression (IHC) C. CD56 expression (IHC) D. SSTR2 expression (IHC) E. INSM1 expression (IHC).

Immunostaining showed that the tumor was neuroendocrine, as they were positive for Chromogranin A (CgA), Synaptophysin (Syn), CD56, CK, CK7, CK8, Epithelial membrane antigen (EMA), Recombinant Somatostatin Receptor 2(SSTR2), insulinoma-associated protein 1(INSM1), KI-67 was 70 %. While the Vim, LCA, WT-1, CA125 were negative. (Fig. 4).

Adjuvant chemotherapy was administered, and the patient tolerated 8 cycles of cisplatin (75 mg/m^2^ on day 1) and etoposide (100 mg/m^2^ on days 1, 2, and 3) well. Eight months after the initial diagnosis, the patient is alive with no evidence of recurrence or relapse. A CT scan of the abdomen (Fig. 2 C-D) indicated a significant effect of the chemotherapy.

**Fig. 5:**
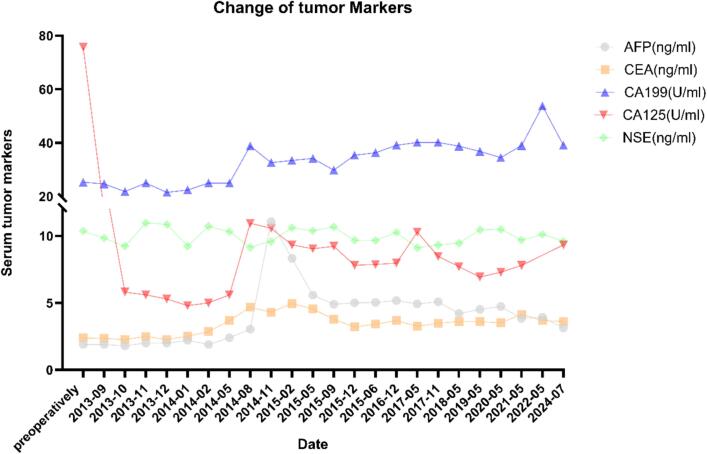
Tumor Marker changes in exceeding 10 years follow-up.

After completing the standard treatment, the patient was followed up according to the routine protocol for gynecological tumors for 10 years, with no recurrence or metastasis observed on imaging. Recent CT shows the retroperitoneal and bilateral iliac vascular lymph nodes, and lung scattered calcified nodules are similar as before (Fig. 2 E-F). Tumor markers are normal. No hormone secretion symptoms were observed. (Fig. 5).

## 3 Comment

Neuroendocrine carcinomas most commonly originate in the digestive system and lungs, but they are rarely found in the female genital tract. There is no literature reporting the incidence and survival rates of tubal NENs. Tubal NEC may originate from Müllerian duct epithelial stem cells. Other potential pathogenic mechanisms include erroneous migration of neuroendocrine cells, implantation during previous surgeries, neuroendocrine differentiation of unanchored stromal cells, or metastasis (Neumann et al., 2010). Diagnosing neuroendocrine carcinomas before surgery is challenging due to the lack of obvious symptoms and specific examinations. Therefore, its diagnosis primarily relies on postoperative pathology at present.

In comparison to ovarian neuroendocrine carcinoma (NEC), which has been described more frequently, primary NEC arising from the fallopian tube remains extremely rare and may present with subtler clinical findings. Although both types of tumors can exhibit small cell or large cell morphology and rely on immunohistochemical markers such as synaptophysin and chromogranin A for diagnosis, their embryologic origins differ (Saraei et al., 2024). When both the tube and ovary appear involved, meticulous pathological examination is essential to identify a primary tubal lesion with secondary ovarian spread. Nonetheless, from a therapeutic standpoint, the treatment approach largely overlaps with that for ovarian NEC, typically emphasizing extensive surgical debulking followed by platinum-based chemotherapy.

According to the ESMO Guidelines (Pavel et al., 2020 Jul 1), functional neuroendocrine tumors may exhibit elevated levels of CgA, NSE, and related peptide or amine hormones. In contrast, non-functional neuroendocrine tumors may not show significant hormone changes. Kwon et al. (Kwon et al., 2023) reported elevated serum chromogranin A levels in Grade 2 fallopian tube NETs. However, no related hormone changes have been observed in other fallopian tube neuroendocrine tumors. Among the emerging biomarkers for neuroendocrine neoplasms (NENs), NETest (Puliani et al., 2022 Aug 5) is relatively well-established, detecting the levels of 51 specific gene transcripts with sensitivity and specificity greater than 90 %. Ultrasound, CT, and MRI are commonly used for localization and diagnosis of lesions. Currently, molecular imaging diagnostics such as SPECT, 18F, and 68 Ga offer higher sensitivity in the early diagnosis and staging of neuroendocrine tumors (Kim et al., 2020 Dec)^.^ In our case, the patient's CA125 levels were elevated, while other tumor markers, including NSE, were normal. This case was limited by the diagnostic capabilities at the time, and only ultrasound and CT scans were conducted. Preoperative chest and upper abdominal CT scans were normal, ruling out the possibility of metastasis of neuroendocrine tumors from the lungs, gastrointestinal tract, or pancreas to the pelvis, suggesting the likelihood of a primary gynecological tumor.

The diagnosis of NEC relies on immunohistochemical analysis. Commonly used immunohistochemical markers include Syn, CgA, EMA, Ki67, SSTR2 (Shenoy, 2024). Among these biomarkers, the most sensitive biomarker is Syn, while CgA is the most specific. In addition, immunohistochemical markers such as, INSM1, CD56, O6-methylguanine-DNA methyltransferase (MGMT), p53, Recombinant Retinoblastoma Protein 1 (RB1), death domain-associated protein (DAXX), and Alpha-Thalassemia/Mental Retardation (ATRX) are also recommended (Nagtegaal et al., 2020) in gastrointestinal neuroendocrine tumors. In a study of cervical NEN, INSM1 was found more specific than normal markers (>95 %) (Hou et al., 2018). In our case, immunostaining showed positive results for CgA, Syn, CD56, SSTR2, INSM1, CK and Ki-67, consistent with the histopathologic examination, confirming the neuroendocrine nature of the tumor. Primary neuroendocrine carcinoma of the fallopian tube is exceedingly rare. However, apart from pathological, immunohistochemical, and radiological findings, there are no other methods or immunomarkers that can effectively distinguish primary NETs in the fallopian tube from metastatic NETs originating from other sites. However, based on preoperative imaging, postoperative pathology, and immunohistochemistry, the clinical diagnosis of primary neuroendocrine tumor of the fallopian tube can been considered.

Gene testing is an emerging and advanced method that holds promise for guiding diagnosis and subsequent treatment. Approximately 5–10 % of NENs are associated with genetic factors, involving germline autosomal dominant mutations in genes such as MEN1, MEN2, MEN4, VHL syndrome, TSC, and NF1. Data on whole-exome sequencing (WES) and whole-genome sequencing (WGS) of NENs are currently very limited. A large-scale genetic study (van Riet et al., 2021 Jul 29) identified somatic mutations in genes like RB1, KRAS, TP53, and high tumor mutational burden (TMB) in some advanced NENs, which may offer new treatment options in the future. Liquid biopsy using circulating tumor DNA could become an alternative for molecular tumor analysis in gastrointestinal NEC patients (Sorbye et al., 2023 Mar). Due to the diagnostic limitations at the time, no genetic testing was performed in our pathology. There is also no genetic testing information available for fallopian tube neuroendocrine tumors at present. In future diagnosis and treatment, relevant testing could be conducted to aid in clinical decision-making.

The treatment approaches for NET and NEC differ to some extent. Surgery is the first-line treatment for both types of NEN. In the treatment of NEC, chemotherapy is recommended after surgery to prevent postoperative recurrence. Platinum-based chemotherapy (cisplatin or carboplatin and etoposide [EP/EC] or cisplatin and Irinotecan [IP]) is the standard first-line treatment (Expert Committee on Neuroendocrine Neoplasms, Chinese Society of Clinical Oncology. Chinese expert consensus on gastroenteropancreatic neuroendocrine neoplasms, 2022). However, no effective drug therapy has been established for cases refractory to these therapies. What’s more, targeted therapy drugs (including mTOR inhibitors and tyrosine kinase inhibitors), immune checkpoint inhibitors (such as nivolumab and pembrolizumab), and radionuclide therapy are indicated (Xu and Li, 2024) for further control of tumors. There is currently no clinically effective data for immune checkpoint inhibitors, and they are only used to treat a subset of patients who still have metastases after multiple lines of therapy. We performed an open hysterectomy with bilateral salpingo-oophorectomy and omentectomy Following the treatment guidelines for gastrointestinal neuroendocrine tumors, the patient underwent 8 cycles of cisplatin (75 mg/m^2^ on day 1) and etoposide (100 mg/m^2^ on days 1, 2, and 3), and significant therapeutic effects were observed. Summarize previous cases for fallopian tube NECs, adjuvant therapy primarily involves carboplatin and etoposide, some supplemented with paclitaxel, vincristine, and Adriamycin. Currently, no adjuvant therapy was report for fallopian tube NET. Kwon et al. (Kwon et al., 2023) report the first case for using somatostatin analogue therapy on Grade 2 NETs in stage IIIAi patients, though its effectiveness is currently unknown.

The follow-up for patients with neuroendocrine tumors is related to the tumor’s grade, stage, and functionality, specifically every 3 months for the first 2 years, semi-annually for the 3rd to 5th years, and annually after 5 years. Follow-up typically involves monitoring tumor progression and managing symptoms related to hormone secretion in functional tumors, including clinical symptoms, biochemical markers, and routine imaging such as contrast-enhanced CT or MRI of the abdomen and pelvis. Additional imaging, such as somatostatin receptor scintigraphy, PET/CT or PET/MRI, and FDG-PET/CT, can be performed based on clinical needs (Ito et al., 2021). In this case, tumor markers, contrast-enhanced CT, and MRI evaluations were conducted according to the standard timeline for gynecologic malignancies. The patient exhibited no significant hormone-related symptoms preoperatively and did not develop any postoperative symptoms. Routine assessments over 10 years revealed no recurrence and endocrine function.

Although small cell neuroendocrine carcinoma (NEC) of the female genital tract generally has a poor prognosis, our patient remained disease-free for more than a decade. Several factors may account for this favorable outcome. First, thorough surgical cytoreduction (total hysterectomy, bilateral salpingo-oophorectomy, and omentectomy) presumably minimized residual disease burden. Second, small cell NECs often demonstrate notable sensitivity to platinum-based chemotherapy, and our patient was able to tolerate eight full cycles of cisplatin plus etoposide, which may have eradicated microscopic tumor foci. Third, aside from controlled hypertension, she had no significant comorbidities, allowing for strict adherence to follow-up imaging and early management of any potential recurrence. Lastly, although no genetic or molecular profiling was performed, the possibility of favorable molecular alterations cannot be discounted. Future large-scale genetic studies on similar cases may clarify the underlying reasons for such prolonged survival.

In conclusion, neuroendocrine carcinomas can arise from various sites within the female genital tract, including the fallopian tubes. Our case showed that after the radical tumor surgery, the combination of cisplatin and etoposide in eight rounds of chemotherapy has demonstrated the effective anti-tumor efficacy for the longest survival time of more than 10 years. Guidelines for the diagnosis and treatment of female reproductive system NEN should be established through the comprehensive analysis of additional case data, aiming to achieve much more effective and precise diagnosis and treatment.

## Author Contributions

Jun Ma collected basic information from the patient and image data, and wrote the main content of the manuscript; Yujing Tang revised the language of the manuscript and helped write it; Wei He completed the pathological analysis. Jun Shi provided guidance on the writing of the manuscript. All authors have read and agreed to the published version of the manuscript.

## CRediT authorship contribution statement

**Jun Ma:** Writing – original draft, Data curation. **Yujing Tang:** Writing – original draft. **Wei He:** Formal analysis. **Jun Shi:** Writing – original draft, Data curation.

## Declaration of competing interest

The authors declare that they have no known competing financial interests or personal relationships that could have appeared to influence the work reported in this paper.
